# Respiratory Symptoms and Pulmonary Function of Workers in the Waste Management Industry

**DOI:** 10.7759/cureus.17027

**Published:** 2021-08-09

**Authors:** Chrysovalantis V Papageorgiou, Petros Savourdos, Eleni Douna, Vasiliki E Georgakopoulou, Sotiria Makrodimitri, Georgios Dounias

**Affiliations:** 1 Pulmonology, Laiko General Hospital, Athens, GRC; 2 Department of Occupational & Industrial Hygiene, National School of Public Health, Athens, GRC; 3 Pulmonology, Sismanogleio Hospital, Athens, GRC

**Keywords:** waste management, respiratory symptoms, pulmonary function, spirometry, occupational health hazards

## Abstract

Introduction: Waste handling workers are exposed to air pollutants and toxic compounds produced during waste management and processing that can cause respiratory symptoms and lung function impairment. This study aimed to evaluate the respiratory health of exposed workers in a waste management plant in Attica, Greece.

Methods: 50 field workers exposed to outdoor pollutants (exposure group) and 32 office clerks with no exposure (control group) were evaluated. Upper and lower respiratory symptoms were documented and spirometry was performed.

Results: There was no statistically significant difference between the exposure and the control group in forced expiratory volume in one second (FEV1)%, forced vital capacity (FVC)%, FEV1/FVC% predicted values. Workers had lower maximal mid-expiratory flow (MMEF)% predicted values compared to controls (82% vs 94%, p=0.019). No difference was observed regarding the respiratory symptoms between the two groups.

Conclusion: Lower MMEF values were observed in the exposure group. Low MMEF can be indicative of small airway disease, thus smoking cessation, close follow-up, and the use of personal protective equipment are recommended.

## Introduction

Municipal solid waste management is an issue with both economic and environmental implications. The waste and recycling sector is growing, and with it, public health concerns are rising with the operation of these facilities especially for those working at landfill sites and in waste management plants, and also for the population residing in the vicinity of such plants.

Workers in the solid waste management industry are exposed to numerous occupational risks, arising both from the use of mechanical and electrical equipment and the exposure to toxic compounds produced during waste management and processing [1]. Decomposition of organic waste produces bioaerosols that contain endotoxins, beta-glucans, bacteria, and fungi, which are potent inflammatory agents that induce inflammation in the workers’ respiratory tract [2-4]. Exposure to bioaerosols increases the risk of chronic obstructive pulmonary disease (COPD) [5] and the repeated inhalation of and sensitization to these organic compounds may result in hypersensitivity pneumonitis [6].

Additionally, the increased physical effort of solid waste workers leads to increased ventilation, thus increasing not only the amount of inhaled organic compounds but also the inhalation of dust and exhaust fumes. Bad weather conditions and extreme temperatures may also contribute to the exacerbation of underlying respiratory conditions such as asthma and COPD. Upper respiratory symptoms such as rhinorrhea, nasal congestion, sneezing, and sore throat and lower respiratory symptoms such as cough, sputum production, wheezing, and dyspnea are commonly documented in solid waste workers [7-10].

Therefore, the respiratory health of solid waste workers must be routinely monitored. Lung function tests are an integral part of respiratory system evaluation and should be implemented via a comprehensive workplace respiratory health program [11]. Landfill workers appear to have impaired spirometry results compared to the control group, and restriction pattern seems to be the commonest type of impairment [9]. Exposure to organic dust has been associated with a long-term decline in forced expiratory volume in one second (FEV1) among the exposed in comparison to those of the control group [12], while a decrease in lung function parameters was observed in workers exposed to waste-to-energy plants [13], or municipal solid waste workers [10,14].

This study aimed to investigate the respiratory health of field workers in the waste management industry compared to a group of office clerks working in the same facilities, with no occupational exposure to outdoor pollutants, as reflected in the severity of symptoms and the results of lung function tests.

## Materials and methods

Study population

The study was conducted in the solid waste management facility of Attica, Greece which is located 20km from the capital Athens, and located between the mountains Parnitha and Aigaleo. It comprises a sanitary landfill where waste is deposited and buried in a designated area of 1km2, recycling and composting plant, a healthcare waste incineration facility, and sewage treatment and biogas energy production facility, with corresponding administrative offices located in the same area. It receives and manages the total waste production in the broader area of Attica with a population of four million people, and employs 438 people of different specialties.

In the present study, we randomly selected 50 field workers with more than five years of working time at the waste management plants (exposure group), and 32 office clerks with the same duration of working time at the same facilities with no exposure to outdoor pollutants (control group), proportionally from all working sites in the plant. All recruited individuals had no prior medical history of respiratory diseases. All participants gave their informed consent. The study protocol was approved by the National School of Public Health ethics committee (approval no: 3288/13.3.2018).

Symptom evaluation

The subjects were interviewed using a structured questionnaire. Demographic data, personal and occupational history, and smoking status were recorded. Symptoms of the upper airway were assessed by recording common complaints such as nasal congestion, running nose, sneezing, and sore throat. A slightly modified version of the Medical Research Council (MRC) questionnaire [15] was used to evaluate lower respiratory symptoms such as cough, phlegm production, wheezing, and dyspnea.

Spirometry

Spirometry was performed using a portable spirometer (SpiroAnalyzer ST-150, Fukuda Sangyo, Japan), in a sitting position, according to the joint European Respiratory Society (ERS)/American Thoracic Society (ATS) clinical practice guidelines [16]. At least three acceptable FVC maneuvers were performed and the best values of FEV1 and forced vital capacity (FVC) were recorded. Tiffeneau index (FEV1/FVC) and maximal mid-expiratory flow (MMEF) were also recorded based on the maneuver with the best sum of FEV1 and FVC. All parameters were expressed as absolute values and as a percentage of the predicted value based on sex, age, height, and ethnicity.

Statistical analysis

Statistical analysis was performed using Sigmaplot v11 statistical software (Systat Software, Inc., San Jose, CA, USA). Comparisons between groups for continuous variables were performed using t-test for normally distributed data and Mann-Whitney test for not normally distributed data. Comparisons between groups for categorical variables were performed using the chi-square test and Fisher exact test. Multiple logistic regression was used to adjust for smoking status. A p-value of 0.05 was considered significant.

## Results

Demographic characteristics, smoking status, and working time data of the two groups are summarized in Table 1.

**Table 1:** Demographic characteristics, smoking status, and working time data of the two groups. Values are expressed as mean±SD

Demographic Characteristics	Workers (n=50)	Controls (n=32)	P-value
Gender			<0.001
Male	46 (92%)	18 (56%)	
Female	4 (8%)	14 (44%)	
Age (years)	48±9.3	45±7.1	0.091
Height (cm)	176±8	171±7.9	0.008
Weight (kg)	85±14.5	88±24	0.746
Smoking Status			0.554
Current	26 (52%)	13 (40.5%)	
Ex	9 (18%)	6 (19%)	
Non-smokers	15 (30%)	13 (40.5%)	
Working Time			0.985
5-10 years	18 (36%)	13 (41%)	
10-15 years	17 (34%)	11 (34%)	
15-20 years	11 (22%)	6 (19%)	
>20 years	4 (8%)	2 (6%)	

The exposure group had a higher percentage of males (92% vs 56%, p<0.001) and taller height (176cm vs 171cm, p<0.01) compared to controls. No statistically significant differences were observed regarding age, weight, smoking status, and working time between the two groups. Although the use of protective equipment (masks, gloves, working uniforms) is strongly recommended, 16 workers (28%) never used respiratory protective equipment and 13 workers (26%) used it occasionally.

Spirometry was performed in all participants and acceptable FVC maneuvers were obtained by all individuals. Seventy-six percent of the workers and 87.5% of the office clerks had normal spirometry. A mild or moderate restrictive pattern was observed in 20% of the workers and 12.5% of the controls. The obstructive pattern was observed only in two participants of the exposure group (one worker with mild, and the other with severe disease). The spirometry results and the spirometric values expressed as a percentage of the predicted are summarized in Table 2.

**Table 2:** Spirometry results and spirometric values (expressed as % of the predicted) of the two groups (mean±SD).

Spirometric Results	Workers	Controls	P-value
Normal spirometry	38 (76%)	28 (87.5%)	0.4
Obstructive pattern Mild Moderate Severe	2 (4%) 1 1	0	
Restrictive pattern Mild Moderate Severe	10 (20%) 4 6 0	4 (12.5%) 3 1 0	
FEV1 % pred	87±15.9	92±10.5	0.159
FVC % pred	86±16.1	90±9.8	0.252
FEV1/FVC % pred	104±7.9	107±5.6	0.075
MMEF % pred	82±23	94±22	0.019

There was no statistically significant difference between the workers’ group and the control group in FEV1%, FVC%, FEV1/FVC% predicted values. Lower MMEF% predicted values were observed in the workers’ group compared to controls (82% vs 94%, p=0.019). After adjustment for smoking status, the difference between the two groups remained statistically significant.

Respiratory symptoms reported by the two groups are listed in Table 3.

**Table 3:** Respiratory symptoms reported by the two groups.

Respiratory Symptoms	Workers	Controls	P-value
Upper Airway Symptoms	5 (10%)	6 (18%)	0.325
rhinorrhea	3	4	
nasal congestion	3	2	
sneezing	3	3	
sore-throat	1	1	
Lower Airway Symptoms	13 (26%)	9 (28%)	0.965
cough	11	7	
phlegm production	4	3	
wheezing	1	0	
dyspnea	2	2	

Five workers and six office clerks reported at least one upper airway symptom, whereas 13 workers and nine clerks reported at least one lower respiratory symptom. There was no statistically significant difference between the two groups in the proportions of the subjects who reported symptoms of the upper (10% vs 18%, p=0.325) and the lower respiratory tract (26% vs 28%, p=0.965).

## Discussion

This is a cross-sectional study that investigated the respiratory health of field workers in the solid waste management industry, as reflected in respiratory symptoms and spirometric measurements, in comparison to office clerks who were not exposed to outdoor pollutants. Impaired spirometric values were observed in 24% of the workers and 12.5% of the clerks and restriction was the pattern that was observed in the majority of the cases. Additionally, after adjusting for smoking status, workers had lower MMEF% predicted values in comparison to the control group. Mid-expiratory flow is a surrogate for small airway disease and that is why close monitoring of the worker's respiratory health and the implementation of smoking cessation programs are extremely important.

In our study, restriction was the main pattern observed in individuals with impaired spirometry. This is in accordance with the findings of Ray et al., who examined workers employed in a municipal solid waste disposal landfill site in Delhi, and observed that 42% of the workers had a restrictive pattern in spirometry [9]. Hamid et al. assessed the respiratory health of elementary workers and observed that 46% of solid waste pickers had restrictions in spirometry, attributing this finding to the possible weakness of lung muscles, obesity, scoliosis, or stiffness of the chest wall [17]. Van Kampen et al. observed that although spirometry results of compost workers were within normal limits, FVC% predicted values were significantly lower compared to the controls [18]. Similar findings were observed in the study of Athanassiou et al. where FVC% predicted values of 104 municipal solid waste workers were significantly lower compared to the controls [10]. In our study, the percentage of workers with restrictive spirometry was lower. However, since static lung volumes were not measured by body plethesmography or by multi-breath nitrogen washout in any study, the diagnosis of restriction cannot be unequivocally verified.

In the present study, there was no difference in FEV1% predicted values between the workers and the control group. This is in accordance with the findings of van Kampen et al, who detected no difference in FEV1% predicted values between compost workers and controls [18]. Coppeta et al. studied workers in a waste-to-energy plant and observed no significant difference in lung function parameters between low and high exposure groups after a five-year exposure period [13]. Bresnitz et al. noted that high and low exposure groups of waste incinerator workers had similar spirometry patterns with no significant differences in FEV1% predicted and FVC% predicted values [19].

On the contrary, Vimercati et al., who examined the respiratory health of 124 waste collection and disposal workers in Italy, observed lower mean FEV1 values in the exposed workers compared to controls [14]. Similar findings were observed in the study of Ray et al. where landfill workers had significantly reduced mean FEV1 values compared to controls [9]. Heldal et al. observed a significant decline in FEV1% predicted values during the working week of waste handlers [2]. The obstructive pattern observed in previous studies has been attributed to the inflammatory response of the airway as a result of bioaerosol exposure and the consequent contact with bacterial endotoxins and beta-glucans.

The maximum mid-expiratory flow (MMEF) of solid waste workers has not been studied as extensively as the other spirometry indices and its report in the literature is limited. Coppeta et al. reported a significant lowering in MMEF% predicted values in exposed waste-to-energy plant workers, which was higher in the more exposed group [13]. Bresnitz et al. investigated the respiratory health of municipal waste incinerator workers and noted that the high exposure group had almost twice as many workers with spirometric changes suggestive of small airway disease, compared to the low exposure group [19]. Similar findings regarding incinerator workers were observed in the study of Charbotel et al., who reported that after a three-year follow-up period, MMEF% predicted values of exposed workers were significantly lower than in the non-exposed group [20]. On the contrary, Aghaei et al. found no difference in MMEF values between exposed workers and office clerks in composting facilities [21]. In our study, workers had lower MMEF values compared to controls. Mid-expiratory flow is considered a test of ventilation sensitive to expiratory flow obstruction and it has been used as a widely available methodology to assess small airways function. Small airways dysfunction may precede airflow obstruction and therefore provide a signal of early obstructive lung disease [22]. Identifying workers at risk can lead to the early implementation of interventions such as close follow-up and smoking cessation counseling.

In our study, no difference was detected in respiratory symptoms between the exposed and the control group. This is in accordance with the findings of Tschopp et al., where respiratory symptoms were not associated with occupational exposure of garbage and wastewater workers [23]. No statistically significant association between occupational exposure and upper and lower airway symptoms was detected in the study of Vimercati et al., and that is why altered respiratory function parameters were considered an early indicator of respiratory disease prior to the onset of respiratory symptoms [14]. On the contrary, a higher prevalence of respiratory symptoms in exposure versus the control group was reported in the study of Athanassiou et al. where municipal solid waste workers suffered more commonly from sore throat, cough, and phlegm production compared to office clerks [10]. Schantora et al. noted that the duration of employment in waste collection was associated with an increased prevalence of rhinitis, cough, and phlegm production [7]. Increased prevalence of cough was also reported in composting plants [21]; exposure to bioaerosols in these facilities has been correlated to irritation of the nose and to respiratory symptoms, indicating the development of chronic inflammation in the upper and lower airway [24]. Waste collectors in Taiwan were at risk of developing chronic respiratory symptoms such as cough, phlegm production, and wheezing compared to controls [25].

A limitation of the present study is that lung function measurements were performed with a portable spirometer on the working site, and thus an objective measurement of static lung volumes could not be performed; the diagnosis of restriction was based on low FVC values since total lung capacity could not be estimated. Additionally, small airway dysfunction was based on low MMEF values. However, MMEF requires cautious interpretation since it is highly effort dependent and the normal range is wide. On the contrary, oscillometry can more reliably detect small airway disease, since it is a technique specific to small airway function, it is effort independent and has a robust normal range [22]. However, it requires specialized equipment not widely available. Future studies could evaluate the respiratory health of solid waste workers in lung function laboratories, where all tests could be performed and objective measurements for all parameters could be obtained.

Another limitation of the study is the small sample size selected from different working sites with variable exposure to air pollutants. However, it is representative of the workers in the waste management plant since it comprises individuals who were randomly selected proportionally from all working sites. Field workers were compared to office employees of the same plant with spirometry measurements and respiratory symptoms. Nevertheless, the concentrations of air pollutants such as inhalable dust, endotoxins, and beta-glucans that the recruited individuals were exposed to were not measured, so the potentially inflammatory effect on the airway in different working sites cannot be estimated. Finally, this is an occupational study of waste handling workers, and the fact that no significant impairment in spirometry measurements was detected could be the result of the healthy worker effect, which is commonly described in similar studies [26,27].

## Conclusions

The spirometric evaluation of waste handling workers detected a significant reduction in MMEF values compared to controls. No significant differences were found regarding the other spirometry indices and the respiratory symptoms. The findings of the present study suggest that the respiratory health of workers in the waste management industry should be regularly monitored, smoking cessation counseling should be implemented and the use of personal protective equipment should be strongly recommended.
